# Causality of small and large intestinal microbiota in weight regulation and insulin resistance

**DOI:** 10.1016/j.molmet.2016.06.002

**Published:** 2016-06-10

**Authors:** Torsten P.M. Scheithauer, Geesje M. Dallinga-Thie, Willem M. de Vos, Max Nieuwdorp, Daniël H. van Raalte

**Affiliations:** 1 Department of Vascular Medicine, Academic Medical Center (AMC), University of Amsterdam, Amsterdam, The Netherlands; 2 Diabetes Center, Department of Internal Medicine, VU University Medical Center, Amsterdam, The Netherlands; 3 Institute for Cardiovascular Research (ICaR), VU University Medical Center, Amsterdam, The Netherlands; 4 WU Agrotechnology and Food Sciences, Wagening University, Wageningen, The Netherlands

**Keywords:** Gut microbiota, Obesity, Diabetes, Weight regulation, Insulin resistance, 16s rRNA, 16S ribosomal RNA (30S small subunit of prokaryotic ribosomes), AMP, adenosine monophosphate, AMPK, AMP-activated protein kinase, Angptl4, Angiopoietin-like 4, AS160, Akt substrate of 160 kDa, CB1R, cannabinoid receptor type 1, CCL2, Chemokine (C–C motif) ligand 2, DIO, diet-induced obesity, GF, germ-free, GLP, glucagon-like peptide, Gpr, G-protein coupled receptor, HFD, high fat diet, IL, interleukin, IRS-1, insulin receptor substrate 1, JNK, C-Jun N-terminal kinase, LBP, LPS-binding protein, LPL, lipoprotein lipase, LPS, lipopolysaccharide, MCP-1, monocyte chemotactic protein 1, NOD1, nucleotide-binding oligomerization domain-containing protein 1, PKB, protein kinase B (also known as Akt), PYY, peptide YY (for tyrosine–tyrosine), RYGB, Roux-en-Y gastric bypass, SCFA, short-chain fatty acid, T2D, Type 2 *diabetes mellitus*, TLR, toll-like receptor, TNF-α, tumor necrosis factor alpha, VLDL, very low density lipoprotein, WHO, World Health Organization, ZO, zonula occludens

## Abstract

**Objective:**

The twin pandemics of obesity and Type 2 diabetes (T2D) are a global challenge for health care systems. Changes in the environment, behavior, diet, and lifestyle during the last decades are considered the major causes. A Western diet, which is rich in saturated fat and simple sugars, may lead to changes in gut microbial composition and physiology, which have recently been linked to the development of metabolic diseases.

**Methods:**

We will discuss evidence that demonstrates the influence of the small and large intestinal microbiota on weight regulation and the development of insulin resistance, based on literature search.

**Results:**

Altered large intestinal microbial composition may promote obesity by increasing energy harvest through specialized gut microbes. In both large and small intestine, microbial alterations may increase gut permeability that facilitates the translocation of whole bacteria or endotoxic bacterial components into metabolic active tissues. Moreover, changed microbial communities may affect the production of satiety-inducing signals. Finally, bacterial metabolic products, such as short chain fatty acids (SCFAs) and their relative ratios, may be causal in disturbed immune and metabolic signaling, notably in the small intestine where the surface is large. The function of these organs (adipose tissue, brain, liver, muscle, pancreas) may be disturbed by the induction of low-grade inflammation, contributing to insulin resistance.

**Conclusions:**

Interventions aimed to restoring gut microbial homeostasis, such as ingestion of specific fibers or therapeutic microbes, are promising strategies to reduce insulin resistance and the related metabolic abnormalities in obesity, metabolic syndrome, and type 2 diabetes. This article is part of a special issue on microbiota.

## Highlights

• The small and large intestinal microbiota influence weight regulation and glucose homeostasis.
• A Western diet, which is rich in saturated fat and simple sugars, may lead to changes in the gut microbial physiology.
• Changes in the gut physiology and microbial composition are involved in the development of metabolic diseases.
• Ingestion of pre- or probiotics and fecal microbiota transplantation may stop this deleterious process.

## 1 Introduction

The ‘twin epidemic’ of obesity and Type 2 diabetes mellitus (T2D) is a global challenge for healthcare [1], [2], [3], [4]. According to the World Health Organization (WHO), 13% of adults were obese and 9% had (undiagnosed) T2D [5], [6], [7]. T2D was estimated to be the 8th leading cause of death in 2015, which will rise to rank 5 by 2030 [8], [9]. These somber perspectives demand an increase in our understanding of the underlying pathobiology.

During the last decade, the gut microbiota has received much attention as a new factor contributing to the pathobiology of metabolic diseases [10], [11], [12], [13]. The term ‘microbiota’ collectively describes all microorganisms on and in the human body (skin, gut, and other tissues). The majority of the up to 100 trillion (10^14^) microbes reside in the colon (10^11^ cells/mL) [14], where they exert numerous functions such as nutrient metabolism, xenobiotic metabolism, maintenance of gut barrier function, development of the gastrointestinal immune system, and protection against pathogens [15], [16], [17].

**Figure 1:**
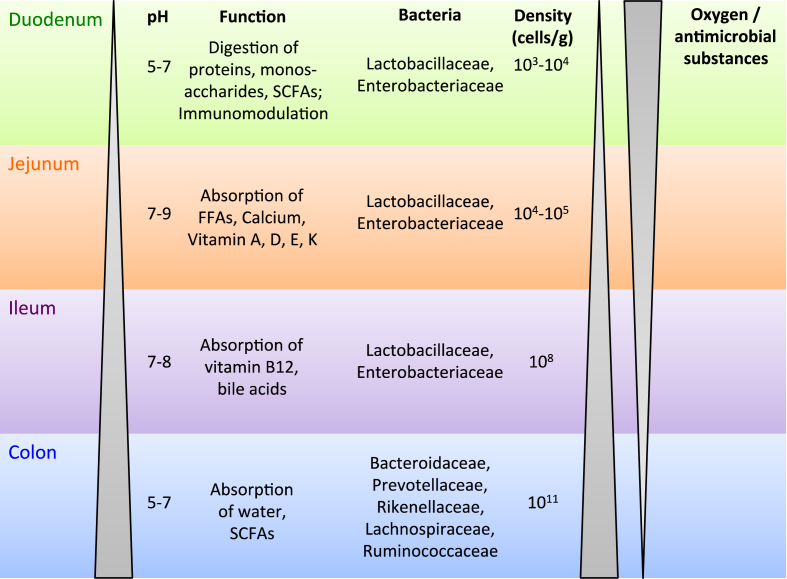
**Function, dominant bacteria, microbial density and oxygen pressure in the different segments of the human intestine**. The pH of the intestine increases from the stomach (1.5–5) to the large intestine (5–7). Similarly, the bacterial density increases from 10^2^ to 10^11^ cells/mL. The small intestine consists of duodenum, jejunum and ileum. Each segment shows different functions, which are mainly responsible for nutrient digestion and absorption. The colon (large intestine) is responsible for absorption of water and fermentation products such as short chain fatty acids (SCFAs). A decrease in oxygen concentration and antimicrobial compounds along the intestine leads to an increasing diversity in the large intestine with several obligate anaerobic bacteria. In upper parts reside more facultative aerobic bacteria, which can tolerate oxygen. Abbreviations: FFA, free fatty acids.

The microbial density increases from the stomach to the rectum (Figure 1), with low densities in the small intestine (10^3^–10^8^ cells/g feces) compared to the large intestine (10^11^ cells/g feces) [18]. This gradient is due mainly to a rapid luminal flow, a low pH in the upper GI tract, and the secretion of anti-bacterial substances such as bile acids [19]. So far, 1057 species have been identified and cultured in the human microbiota, of which each individual harbors at least 160 different species [15], [20]. A high inter-individual variability in the fecal microbial composition makes it difficult to find compositional shifts. A study concluded that it takes at least 535 subjects to conclude significant shifts between groups (e.g. lean and obese) with enough power [21].

Bacteria represent the major part of the indwelling microorganisms compared to virus, fungi, archea, phages, and protozoa. The main bacterial phyla, a high-level taxonomic rank, of the intestinal microbiota are Firmicutes, Bacteroidetes, Actinobacteria, Proteobacteria, and Verrucomicrobia [20]. In healthy conditions, the composition of the microbiome is highly diverse, the microorganisms live in symbiosis with the host and seem to remain rather stable during live-time [22]. The microbial composition is dependent on environmental (for example mode of delivery, breast feeding, diet, medication use, personal hygiene and presence of toxins) and genetic factors [17], [23], [24], [25], [26]. Interestingly, recently it has been found that at least 50–60% of the bacterial genera from the intestinal microbiota produce resilient spores, which makes them more stable in an oxygen rich atmosphere and more likely to be passed from individual to individual [27].

The microbial composition and diversity differ along the intestine [28], [29]. The stomach and small intestine are enriched in Firmicutes (Lactobacillaceae) and Proteobacteria (Enterobacteriaceae), whereas the large intestine shows a higher portion of Bacteroidetes (Bacteroidaceae, Prevotellaceae, Rikenellaceae) and Firmicutes (Lachnospiraceae, Ruminococcaceae), while the sole representative of the Verrucomicrobia, *Akkermansia muciniphila*, is also mainly located here [30].

This difference is due mainly to higher oxygen concentrations and the presence of antimicrobial compounds in the proximal intestine [31], [32], [33]. Many microbial studies have focused on the large intestine and fecal microbiota, while the small intestine microbiota also may have a profound impact on the host's physiology since the small intestine is the first site of contact of the intestine with nutrients, orally administrated drugs, and potential pathogens. More work is needed to elucidate the composition and function of small intestinal microbiota, for which only one comprehensive metagenomic study has been reported [34].

As indicated above, gut microbiota serves important functions in human physiology. Microbiota residing in the large intestinal facilitate fermentation of dietary fibers, which increases the energy harvest by the host from the diet and is an important factor contributing to the growth and maintenance of the gut microbiota. In addition, gut microbiota produces the short-chain fatty acids (SCFAs), such as acetate, propionate, and butyrate, as well the gases carbon dioxide, hydrogen, and methane [35], [36]. SCFAs are quickly absorbed in the colon and used by the host for different purposes. For example, butyrate is mainly used as an energy source for colonic epithelial cells and, at low doses, improves the intestinal barrier function [37] but has also been implicated in regulation of (murine) insulin sensitivity [38]. In contrast, acetate can be used in lipogenesis and propionate in hepatic as well as intestinal gluconeogenesis [10], [39], [40]. Further, gut microbiota has been shown to deliver vitamins to the host such as folates, vitamin K, biotin, riboflavin (B2), cobalamin (B12), and possibly other B vitamins. Thereby, they contribute to the health of the host [41].

The gut microbiota not only metabolizes nutrients but also is involved in the breakdown of several xenobiotics such as drugs, dietary compounds (which are non-digestible for the host), and environmental toxins [42], [43]. Via direct (microbiota-mediated) or indirect mechanisms (host-mediated, but microbiota modified), the gut microbiota can activate or inactivate xenobiotics or even transform them into toxic metabolites. Thereby, the ingestion of these compounds can shape the microbiota composition through antimicrobial activity or selectively enhance the growth specific bacteria. An important example is the transformation of choline-containing compounds into trimethylamine (TMA), which is further metabolized in trimethylamine N-oxide (TMAO) by the liver. Conflicting results have been reported regarding the role of this molecule as a crucial link in development of atherosclerosis [43], which is most likely driven by differences in diet (meat) consumption in these different cohorts [44].

Although the intestinal microbiota carries out these important functions for the host physiology, it nevertheless poses a threat when breaching the intestinal wall [45]. An intact barrier function of the intestine is important to prevent any uncontrolled translocation of bacteria from the intestine into the host. This barrier function is orchestrated by a variety of cells along the intestinal epithelium. It is built by a single layer of cells organized into crypts and villi, which are necessary to increase the surface for optimal nutrient absorption. The intestinal epithelial cells are responsible for the absorptive function but constitute an important barrier between the gut microbiota and the host. Several specialized cells help to maintain this function through the secretion of gut hormones (K- and L-cells) in concert with the thick layer of mucus secreted by goblet cells. Together with antimicrobial proteins (AMPs) secreted by various types of cells, the specialized cells protect the host from microbial invasion. Behind the epithelial layer, a diverse population of immune cells resides in the lamina propria preventing any microbial penetration. A sensitive regulation is necessary not only to prevent bacterial translocation but also to develop an (innate) immune tolerance against beneficial microbes. Thereby, commensal bacteria (beneficial species) are tolerated in the gut of the host [46]. Interestingly, animal studies have shown that some commensal bacteria can increase the function of the gut barrier, such as *Faecalibacterium prausnitzii* via the induction of junction proteins and reduction of pro-inflammatory cytokines or *A. muciniphila* by endocannabinoid production [47], [48]. A sensitive interplay between host and microbe is necessary to keep the mutualistic nature of the host–microbial relationship. It has been shown that resident bacteria profoundly shape the host immune response. As such, germ-free (GF) mice exhibit an impaired intestinal immune system that takes a long time to recover upon colonization [49], [50].

Intestinal immune cells, especially dendritic cells, sample antigens or whole bacteria from the lumen and carry them (alive) to the mesenteric lymph nodes. Thereby, they induce a protective secretory IgA (sIgA) response, which coats bacteria in the intestinal lumen for immune exclusion (excretion) or controlled uptake to further train the immune system. Similarly, gut epithelial cells release microRNAs (miRNA), which can regulates bacterial gene expression and growth [51]. Further, several bacterial taxa have been shown to induce a specific immune response. For example, the exopolysaccharides of adherent *Bifidobacterium brevis* reduce the production of inflammatory cytokines and thus dampen B cell response [52], [53]; the polysaccharide A (PSA) of *Bacteroides fragilis* [54] and SCFAs produced by many *Clostridium* spp. stimulate the production of the anti-inflammatory interleukin (IL)-10 by regulatory T cells (T_reg_) [55]; lastly, segmented filamentous bacteria (SFB) stimulate the development of T helper cells (TH17) [56], which are important for mucosal immunity to extracellular pathogens. All these mechanism are essential to induce an immune tolerance against these species and to enable a colonization of them in the host gut [32], [45], [57].

Finally, the gut microbiota dampens the growth of potential pathogenic bacteria through several mechanisms: 1) inhibition of pathogenic growth via bacteriocins and SCFAs [58], [59], 2) suppression of virulent factors [60], 3) facilitation of host barrier function through up-regulation of the mucus layer and induction of antimicrobial molecules and (fecal) IgA [61], as well as 4) priming of intestinal immune cells [62], [63]. These mechanisms show that there is a sensitive interplay between the host and the microbes to keep a mutualistic relationship, which can be disturbed by several environmental factors.

## 2 Perturbation of the intestinal microbiota

Diet is a major factor driving the composition and metabolism of the gut microbiota [64], [65]. For example, some non-caloric artificial sweeteners (NAS, e.g. saccharin, sucralose and aspartame) change the composition and function of the gut microbiota leading to the development of glucose intolerance. Treatment with antibiotics abrogated this effect (through depletion of the microbiota), suggesting the involvement of gut microbes in the metabolism of NAS. Further, transfer of NAS-treated microbiota into GF mice led to similar effects. These findings highlight the deleterious potential of NAS of inducing glucose intolerance via the intestinal microbiota [66]. Moreover, it has to be tested if other NAS such as sugar alcohols and stevia have the same effect.

Similarly, commonly used emulsifiers (detergent-like molecules) have been shown to induce low-grade inflammation, obesity, and metabolic syndrome in mice. The compounds changed the gut microbial composition and increased their pro-inflammatory potential. Transfer of emulsifier-treated microbiota into GF mice reproduced this phenotype, suggesting a role of these compounds in these conditions [67]. Lastly, in mice and humans it has been shown that a long-term high-fat diet (HFD) has deleterious effects on the gut physiology leading to changes in the gut microbial composition, characterized by Firmicutes domination [68]. In line, in obese subjects with metabolic syndrome and diabetes, an altered microbial composition was observed, which was characterized by lower bacterial diversity. Importantly, a lower diversity is associated with a poor health status [68]. Nevertheless, these data need to be reproduced in humans in order to evaluate their relevance fur human disease development.

Antibiotics have a major influence not only on their targets (bacteria) [42], [65] but also on the host [42]. For example, antibiotic use at a young age is associated with higher weight gain [69]. However, recent cohort studies were not able to reproduce these data [70]. Reasons can be found in the weak stability of the gut microbiome at a young age. The infant gut is not sterile at birth and is particularly susceptible to the introduction of new species [57]. It can be colonized by either skin microbiota (upon caesarian section) or vaginal microbiota (upon vaginal deliveries) [71]. Interestingly, both procedures lead to different microbiota compositions. Further, C-section delivery is associated with increase risk for immune and metabolic disorders. Exposure to maternal vaginal fluids at birth resolved this issue [71].

A stable microbiota develops from the age of 2–3 years [72]. During this period, environmental influences, such as the use of antibiotics or the diet, have a major influence on the symbiosis of host and microbiota and may lead to a disease state [73]. Indeed, it has been shown that low-dose of antibiotic exposure in young mice led to obesity [74]. Fecal transplantation of the stool of antibiotic-treated mice to GF mice revealed that this metabolic trait was transmissible, leading to increased total body and fat mass in the recipients, highlighting the role of antibiotics, diet and the microbiota in the development of obesity [74]. In humans, the role of oral antibiotic use is less clear, which might be explained by the age at which antibiotics are used [69], [70].

Similar to the dietary intake, several drugs influence the gut microbiota [21]. Interestingly, the most commonly used orally administrated anti-diabetic drug metformin changed the microbial composition [75], [76]. It is known to reduce blood glucose levels by inhibition of liver glucose production [77]. Remarkably, when injected intravenously in mice and rats, the drug did not lower glycemia, suggesting an intestine based mechanism of action. Metformin activates a duodenal AMPK dependent pathway to lower hepatic glucose production [78]. Further, metformin treatment increased the abundance of the mucin-degrading bacterium *Akkermansia* and the number of mucin-producing goblet cells in mice. Oral administration of *Akkermansia muciniphila* to HFD-treated mice without metformin enhanced glucose tolerance and attenuated adipose tissue inflammation by inducing a T_reg_ dependent response [48], [75]. Further, a recent study could show that ingestion of metformin increased the abundance of *Escherichia coli* and the SCFAs production was enhanced [65]. These findings highlight the gut microbiota mediated effect of metformin. However, it is not yet clear which role intestinal Gram-negative bacteria (like *E*. *coli*) play in this context.

## 3 The gut microbiota increases the energy harvest from the diet

An interesting observation that was made in GF mice is that they are resistant to diet-induced obesity (DIO). This coincides with lower levels of pro-inflammatory markers (TNF-α) and improved insulin sensitivity as compared to wild-type mice that are on a HFD [79]. Further, transfer of the gut microbiota from DIO mice into GF mice (conventionalization) led to weight gain and insulin resistance in the recipient mice [68], suggesting a potential causal involvement of the gut microbiota in weight regulation and the development of obesity. However, another study could not reproduce these findings, which is most likely explained by differences in gut microbiota composition between commercially available mouse strains (genotype) as well as differences in bacterial contamination in the different vivaria [80]. Nevertheless, most studies favor a certain level of involvement of the gut microbiota in metabolic homeostasis.

Based on comparison of GF mice and conventional mice, several evolutionary advantages with respect to food intake and immune maturation have been proposed for the presence of gut microbiota. First, the gut microbiota is essential for processing (non-digestible) dietary polysaccharides to monosaccharides, which can be absorbed by the host or further fermented to SCFAs by the microbiota. Subsequently, SCFAs are delivered via the portal vein system to the liver, where they serve as substrates for hepatic gluconeogenesis and *de novo* lipogenesis. This leads to an increase in body weight in conventional mice. To date, GF mice do not produce SCFAs in the gut due to the lack of fermenting microbes [81], [82]. Thus, conventional mice harvest more energy from the diet than GF mice [83], [84]. In humans, this can also be seen as a disadvantage given the prevalence of obesity due to excessive food intake combined with a reduced physical activity [85].

Secondly, the gut microbiota not only enhances energy uptake from the gut but also helps to store calories in adipose tissue. For example, the gut microbiota inhibits the expression of Angiopoietin-like 4 (Angptl4) in enterocytes. Angptl4 is a potent inhibitor of lipoprotein lipase (LPL). LPL is the enzyme responsible for the hydrolysis of triglycerides (TG), enabling the uptake of fatty acids in tissues for use of energy or storage. GF mice have higher levels of Angptl4 and therefore less LPL activity leading to reduced fat storage in adipocytes [84]. Interestingly, mice fed a HFD supplemented with probiotic bacteria *Lactobacillus paracasei* exhibited reduced body fat accompanied with increased circulating levels of Angptl4. This suggests that a specific population of the microbiota can induce the expression of Angptl4. Lack of Angpt4 in mice led to higher weight compared to their wild-type counterparts without any difference in food intake, energy expenditure, or locomotive activity. Therefore, they favor energy (lipid) storage rather than utilization. Further, the lipid content in the stools of Angpt4^−/−^ was decreased, and the luminal pancreatic lipase activity was elevated. Interestingly, recombinant Angptl4 administration resulted in decreased pancreatic lipase activity. Therefore, Angptl4 is able not only to inhibit systemic LPL activity (lipid storage), but also to inhibit luminal LPL (lipid digestion and uptake) [84], [86].

Human data also suggest a link between Angptl4 and obesity. Plasma levels of Angptl4 were higher in twins with a low body mass index (BMI) as compared to obese counterparts, suggesting an important role of Angptl4 in obesity [87]. Another study also found a link between Angptl4 and hypothalamus function in mice. Lower levels of Angptl4 in obese mice resulted in a higher AMP-activated protein kinase (AMPK) activity in the hypothalamus, which resulted in higher food intake and lower energy expenditure [88]. In conclusion, these observations suggest that Angptl4 is an interesting mediator of microbiota-promoted obesity.

AMPK is another player in nutrient metabolism that is influenced by microbiota. AMPK is an important sensor, which senses low energy content in cells (high AMP, low ATP content). To resolve the lack of energy, AMPK induces energy producing processes (fatty acid oxidation) and food intake via the hypothalamus [88]. In detail, it deactivates acetyl-CoA carboxylase (ACC), an important enzyme involved in lipogenesis. Subsequently, lower levels of Malonyl-CoA (intermediate in lipogenesis) lose their potential to inhibit Carnitine-palmitoyl transferase 1 (Cp1), which transfers fatty acids in mitochondria for fatty acid oxidation. Thereby, activated AMPK favors direct utilization of fatty acids as an energy source (fatty acid oxidation in mitochondria) instead of energy for demanding processes (e.g. lipogenesis and sterol synthesis) [89].

AMPK is regulated not only via Angptl4 but also by SCFAs produced by the microbiota. Interestingly, GF mice show increased skeletal muscle and liver AMPK activity compared to conventional mice, favoring energy utilization. A lower energy harvest from the diet due to the lack of fermenting microbes may explain the higher activity of the energy sensing AMPK [83], [84]. Given the above-mentioned beneficial effects of SCFAs, SCFA production in obese subjects was increased compared to lean subjects. This was accompanied by changes in the abundance of several bacteria. For example, obese mice showed higher levels of Firmicutes than Bacteroidetes compared to their lean counterparts [90], [91], [92]. Firmicutes are the main producers of SCFAs resulting in increased SCFA in feces of obese mice [93], [94], [95], [96], [97]. Similar ratios were found in human samples, questioning the causal role of SCFA producing intestinal bacteria in human obesity [98].

The gut microbiome (sum of gut microbial genes) of obese subjects also exhibited more genes responsible for carbohydrate fermentation compared to lean microbiomes, which is in line with the higher SCFA concentration in obese subjects [99]. Theoretically, the physiological response to SCFA is disturbed due to chronically high levels of SCFAs (“The dose makes the poison”, Paracelsus). In line with this idea, treatment of intestinal cell cultures with low doses of butyrate enhanced the intestinal barrier function, whereas higher concentrations were toxic [37]. A higher SCFA production also means a higher energy harvest from the diet. A recent study provided some mechanistic insights into the increased energy harvest in obesity by comparing the microbiome of nine lean, morbidly obese, and post-gastric-bypass surgery subjects. Firmicutes were dominant in normal-weight and obese individuals, and the number of H_2_-producing Prevotellaceae (Bacteroidetes) as well as H_2_-utilizing Methanobacteriales (Archaea) was enriched in obese subjects. The authors postulate that H_2_ transfer between bacterial and archaeal species is an important mechanism for increasing energy uptake in obese subjects. After gastric-bypass surgery, Firmicutes and H_2_-producing bacteria were lower than in obese subjects, accompanied by an increase in Proteobacteria [100]. Therefore, a lower energy extraction from the diet may help contribute to the weight loss effects of bariatric surgery.

In summary, in mouse models of obesity and in obese individuals, an increased potential to extract energy from the diet was observed, amongst others through the production of SCFAs. Controversially, treatment with SCFAs in animal models led to an improved glucose homeostasis and weight loss possibly through AMPK-mediated pathways. Therefore, the role of SCFAs in obesity requires further studies.

## 4 The gut microbiota alters satiety signaling

Recent findings point to the involvement of the microbiota in appetite and satiety signals. The gut expresses several proteins, which are involved in the regulation of food intake, for example peptide YY (PYY), which is expressed in enteroendocrine cells (L-cells). Loss of SCFAs production from microbes in GF mice led to a reduced activation of the SCFA receptor G protein coupled receptor (Gpr) 41 and, subsequently, a decrease in PYY expression. Therefore, GF mice show higher energy intake, but a lower weight gain, due to a higher intestinal transit time and lower hepatic lipogenesis [101]. DIO mice as well as human obese subjects have lower fasting circulating levels of PYY [102], [103], but, at this moment, it is not known whether SCFAs can affect PYY homeostasis in obese subjects.

The incretin glucagon-like peptide 1 (GLP-1) is another satiety inducing protein, which is secreted by the intestine. Ingestion of prebiotics increased the synthesis of GLP-1 and PYY in plasma of rodents and humans and was accompanied by reduced hunger rates [104]. Similarly, SCFAs triggered the secretion of GLP-1 from mixed colonic cultures *in vitro* [105]. Both effects were mediated via Gpr43 and Gpr41. Mice lacking these receptors exhibited reduced SCFA-mediated GLP-1 secretion *in vitro* and *in vivo* as well as an impaired glucose tolerance [105]. Interestingly, a recent study in mice found that Bacteroidetes and Firmicutes might be involved in the regulation of the GLP-1 expression. Depletion of both phyla in DIO mice with antibiotic treatment (vancomycin and bacitracin) led to an augmentation of GLP-1 secretion, which improved glucose metabolism. Interestingly, Proteobacteria dominated the microbiota after antibiotic administration and HFD treatment [106]. These findings highlight the involvement of the microbiota in the regulation of GLP-1 and other satiety inducing hormones, but the exact mechanism in obesity remains to be elucidated.

GLP-2 exerts a similar function as GLP-1. Both gut hormones result from post-translational processing of pro-glucagon in intestinal L-cells. GLP-2 is associated with intestinal proliferation and an improvement of the gut permeability [107]. For example, HFD-fed mice have a lower expression of the barrier proteins zonula occludens (ZO) 1 and lower levels of *Bifidobacteria* [108], [109], [110]. Obese mice treated with probiotics (*Bifidobacterium* spp.) have a higher expression of tight junction/barrier proteins and lower levels of plasma LPS, resulting in improved intestinal permeability. Additionally, the GLP-2 expression in the intestine was enhanced [111]. Similar findings were made in a recent study with rats. Interestingly, treatment with oligofructose (prebiotic) showed different findings than probiotic treatment with *Bifidobacterium animalis*. Prebiotics increased portal GLP-1 levels, whereas probiotics increased GLP-2. However, both compounds improved metabolic parameters (energy intake, weight gain, fat mass, and glucose homeostasis) [112], highlighting that GLP-1 and 2 not only decrease gut permeability but also improve glucose homeostasis. Similar findings were also made in humans, in which the GLP-2 concentration of diabetic subjects inversely correlated with insulin resistance [113]. Another prebiotic treatment with inulin pasta improved the intestinal permeability, measured as zonulin expression, and GLP-2 levels in healthy young subjects [114]. Together, these findings highlight the involvement of the gut microbiota in inducing the expression of gut hormones, which further improves gut permeability and metabolic functions.

Glucose-dependent insulinotropic polypeptide (GIP) is the fourth important gut hormone, which is modulated via the gut microbiota. It is expressed in intestinal K-cells and is an important modulator of energy homeostasis and glucose metabolism. Similar to PYY and GLP, its expression was induced by SCFAs, possibly mediated by Gpr43 and Gpr41 [115]. However, it cannot be excluded that other receptors may be involved since deletion of Gpar41 did not lead to differences after SCFA treatment [115].

Recent findings also suggest the involvement of the gut microbiota in the regulation of the endocannabinoid (eCB) system [116], which is involved in the satiety signaling in the hypothalamus [117]. Obese subjects show high eCB levels [118], which correlate with higher food intake. Blockage of the eCB receptor with an antagonist reduced food intake [119]. Further, eCB have been shown to promote macrophage activation, contribute to insulin resistance through activation of peripheral CB1 receptors (CB1R), and promote beta cell failure [120]. First evidence for the link between specific bacteria and the endocannabinoid system came from a study in which administration of *Lactobacillus acidpophilus* modulated the expression of cannabinoid receptors in intestinal cells in rats [121]. Further, the gut microbiota seems to modulate the intestinal eCB tone. Obese subjects show higher eCB levels, which can be increased via HFD and reduced by prebiotic treatment as well as antibiotic treatment [122]. Chronic CD receptor stimulation induced glucose intolerance, stimulated metabolic inflammation, and altered lipid storage in skeletal muscle [116], [123].

## 5 Involvement of the microbiota in insulin resistance

In their seminal paper, Cani et al. [124] were the first to show that a 4-week HFD in mice led to diet induced obesity (DIO) and insulin resistance. These changes were accompanied with high levels of lipopolysaccharide (LPS) and increased pro-inflammatory markers such as TNF-α, IL-1 and IL-6. LPS is part of the cell membrane of gram-negative bacteria (mainly Bacteroidetes and Proteobacteria), which are commonly found in human and mice gut microbiota. LPS is continually produced due to constant breakdown of intestinal gram-negative bacteria and is able to translocate from the intestine to several tissue sides. When bound to the toll-like receptor (TLR) 4, it triggers a pro-inflammatory response, providing a link between diet, microbiota and metabolic diseases.

A metagenome-wide association study (MGWAS) showed that there are similar microbial aberrations in (pre) diabetic subjects [125]. They found a decrease in the abundance of some universal butyrate-producing bacteria and an increase in several opportunistic pathogens. Interestingly, the ‘anti-inflammatory’ associated strain *F. prausnitzii* showed a decreased abundance, whereas several common infectious and LPS-producing bacteria were enriched, explaining, to some extent, the impaired barrier function and high LPS level in diabetic subjects [125], [126]. Similarly, a decreased colonic level of *A. muciniphila* has been noted in obese subjects [30].

Increased LPS levels in plasma are associated with a low-grade inflammation [127]. TLR 4, which is expressed mainly on innate immune cells, but also several other tissues, mediates the LPS-induced inflammatory response. TLRs are specialized pattern recognition receptors (PRR), which recognize pathogen associated microbial products (PAMPs) such as LPS, thereby inducing an innate immune response to eradicate invading microbes [128]. Interestingly, *Tlr4*^*−/−*^ mice have lower expression of pro-inflammatory markers such as TNF-α and IL-6 and are protected from insulin resistance, emphasizing that inflammation may affect metabolism [109], [129].

Increased plasma LPS levels of diabetic and obese subjects are negatively correlated with muscle insulin sensitivity. LPS treatment of a human muscle cell line increased the expression of pro-inflammatory markers monocyte chemotactic protein 1 (MCP-1) and IL-6 and reduced insulin stimulating factors such as insulin receptor substrate 1 (IRS-1), protein kinase B (Akt), and Akt substrate of 160 kDa (AS160). Inhibition of Tlr4 suppressed this inflammatory response and resolved the insulin resistance [130]. In conclusion, higher levels of pro-inflammatory factors such as LPS leads to inhibition of insulin signaling in several tissues, which may lead to insulin resistance in a chronic condition [131].

Bacterial components such as LPS, but also live bacteria are able to translocate from the intestinal lumen to other tissue sites. For example, a HFD in mice led to translocation of viable intestinal bacteria to adipose tissue where they induce inflammation. This process occurred before the onset of diabetes. Mice lacking the PRRs nucleotide-binding oligomerization domain-containing protein 1 (Nod1) or Tlr4 were protected from the bacterial translocation. The bacterial translocation could be reversed with probiotic treatment of *B. animalis*, which improved the overall inflammatory and metabolic status of the animal. These findings demonstrate that bacterial translocation occurs before the onset of metabolic diseases and is an integral part of the development of these conditions [132]. Similar findings were made in humans, where diabetic subjects had higher blood levels of 16S rDNA than controls. Interestingly, no differences were observed in obese subjects, except those who had abdominal adiposity [133].

Several mechanisms have been proposed to explain bacterial translocation. The internalization of bacteria by enterocytes with an intact barrier function resembles phagocytosis and is Tlr4 dependent. Interestingly, the bacteria remain viable in the phagosome [134]. Further, epithelial cells *in vitro* and *in vivo* were able to internalize LPS-coated latex particles [134]. Similarly, innate immune cells could phagocytize intestinal microbes. For example, green-fluorescent labeled *E. coli* was co-localized with dendritic cells (DC) in the intestinal lamina propria of DIO mice. After phagocytosis, the DCs disseminated into mesenteric lymph nodes (MLN) and mesenteric adipose tissue (MAT) inducing an inflammatory response similar to LPS [132]. So far, translocation of Gram-negative bacteria has been associated with the metabolic disturbances. The strain *Enterobacter cloacae* (B29) was highly abundant in the gut of a morbidly obese subject (35%) with metabolic syndrome features. Upon extensive weight loss *E. cloacae* was not detectable anymore [135]. However, presence of Proteobacteria has to be established in larger studies to prove its abundance in obesity and diabetes. In contrast to *E. cloacae*, an increased abundance of *Bifidobacterium* is associated with a healthy gut microbiota. Obese [95] and diabetic subjects [136] had lower levels of *Bifidobacterium*, which was associated with higher inflammatory markers as well as disturbed glucose homeostasis and lipid metabolism.

**Figure 2:**
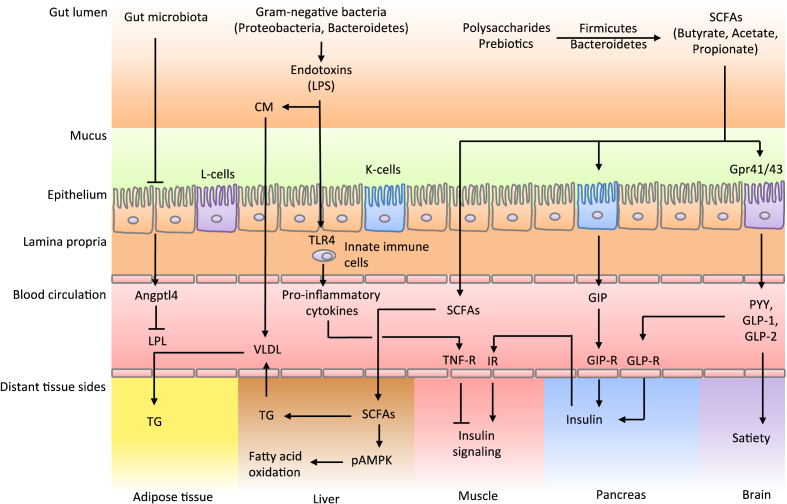
**Involvement of the gut microbiota in weight regulation and insulin resistance**. The gut microbiota is able to ferment polysaccharides into monosaccharides and short-chain-fatty-acids (SCFAs). These products are taken up by the epithelium and transported to the liver. An obese type of microbiota shows higher levels of Firmicutes than Bacteroidetes, which is associated with a higher SCFA production leading to more energy extraction from the diet. Further, the altered microbiota leads to a lower expression of Angiopoietin-like 4 (Angptl4), which inhibits Lipoprotein lipase (LPL) activity. This enzyme facilitates the hydrolysis of triglycerides (TG) in very low-density lipoprotein (VLDL) and chylomicrons resulting in the uptake of fatty acids in skeletal muscle, heart, and adipose tissue. An obese-type microbiota shows higher TG storage in adipocytes. Similarly, obese subjects show lower activities of phosphorylated adenosine monophosphate protein kinase (pAMPK), which is necessary for the activation of fatty acid oxidation. Lastly, an altered microbiota is associated with lower expression of satiety inducing gut hormones such as peptide YY (PYY), glucagon-like peptide (GLP) 1 and 2.

In summary, a connection between the microbiota and the glucose tolerance has been postulated (Figure 2). However, the underlying pathobiology responsible for changes in the gut microbiota leading to obesity and T2D remain elusive. Nevertheless, the gut microbiota is involved in the induction of insulin resistance via promoting a pro-inflammatory response and SCFA production.

## 6 Treatment strategies for obesity and diabetes

Several treatment strategies targeting the gut microbiota have been tested. Probiotics may help to restore microbiota function. As mentioned before, patients with diabetes show lower levels of *Bifidobacterium* spp. and *F. prausnitzii*, which are Gram-positive bacteria that are associated with anti-inflammatory properties [137], [138]. Ingestion of probiotic yogurt containing *Lactobacillus acidophilus* and *Bifidobacterium lactis* for 6 weeks decreased fasting blood glucose and hemoglobin A1c (HbA1c) in diabetic subjects. Further, it improved antioxidant status [139], [140] and lowered TNF-α levels [141]. In another study, treatment of diabetic subjects with *L. acidophilus* for 4 weeks led to improved insulin sensitivity, but did not alter inflammatory parameters [142]. Treatment with another strain (*Lactobacillus plantarum*) enhanced the intestinal barrier by increasing the expression of ZO-1 and occludin in tight-junction structures [143]. In summary, probiotics are a promising agent for the diabetes management, but further evaluation is required.

Fecal microbiota transplantation (FMT) is another approach to ameliorate the disturbed gut microbiota composition. The first successful findings were made in patients with recurrent *Clostridium difficile* infection with antibiotic resistance and repetitive treatment failure. Infusion of healthy donor feces into infected patients led to an improvement of *C. difficile* associated diarrhea after the first infusion in 81% of the patients. After treatment, patients showed increased fecal bacterial diversity and increased levels of Clostridium clusters IV and XIVa, which are associated with the mucosa health via the production of the SCFA butyrate [144]. Further, a decrease in Proteobacteria, which are generally associated with infections, was observed [145].

The transfer of fecal microbiota from lean donors to obese subjects was similarly successful. Recipients of a lean-type microbiota showed an improvement in insulin sensitivity and higher abundance of butyrate-producing species six weeks after transplantation [146]. Although these clinical findings need to be reproduced, they do show the innovative potential of FMT in treatment of several (metabolic) disease types.

Bariatric surgery (weight loss surgery) is another, more drastic approach to improve metabolic function in (morbid) obesity. Roux-en-Y gastric bypass (RYGB) is the most common weight loss surgery and is performed to decrease the stomach volume, which leads to reduced food intake. Interestingly, improvement in glucose metabolism is independent of weight loss, suggesting other mechanisms may be involved [147], [148]. Bariatric surgery was associated with increased PYY and GLP-1 levels post surgery, explaining, to some degree, the weight reduction due to higher satiety signals [149]. This correlated with increased concentration of fecal and plasma bile acids. Bile acids are ligands of the transmembrane G protein-coupled receptor TGR5, which is expressed on intestinal cells and can induce the expression of GLP-1 and PYY. This explains, to some extent, the higher satiety signal and improved glucose homeostasis [150], [151], [152]. Similarly, binding to the intercellular farnesoid X receptor (FXR) increased beta-oxidation and decreased lipogenesis [153]. Interestingly, DIO mice lacking FXR show a reduction in the beneficial effects of the surgery, highlighting the importance of this receptor in bariatric effects [154]. Indeed, smaller studies in humans have suggested that bariatric surgery was able to change the gut microbial composition. For example, *F. prausnitzii* negatively correlated with inflammatory parameters, strengthening the association of this species as an anti-inflammatory commensal bacterium [138], [155]. However, Proteobacteria increased and Firmicutes decreased after the procedure. Importantly, Proteobacteria represent mostly facultative anaerobes, compared to obligate anaerobic dominating Firmicutes. An increase of dissolved oxygen after RYGB surgery might result in the increase Proteobacteria [156], as has been proposed for ileostomic surgery [157]. Further, gastric acid production is decreased after surgery leading to a higher pH in colonic lumen. pH-sensitive bacteria from the Bacteroidetes and Firmicutes phyla may be inhibited by a higher pH [59]. An increase of Proteobacteria may include a potential risk for bowel inflammation due to its pathogen traits [158]. In summary, bariatric surgery not only changes the intestinal physiology but also the microbial composition with some potential beneficial traits for the host.

## 7 Conclusion and future perspectives

**Figure 3:**
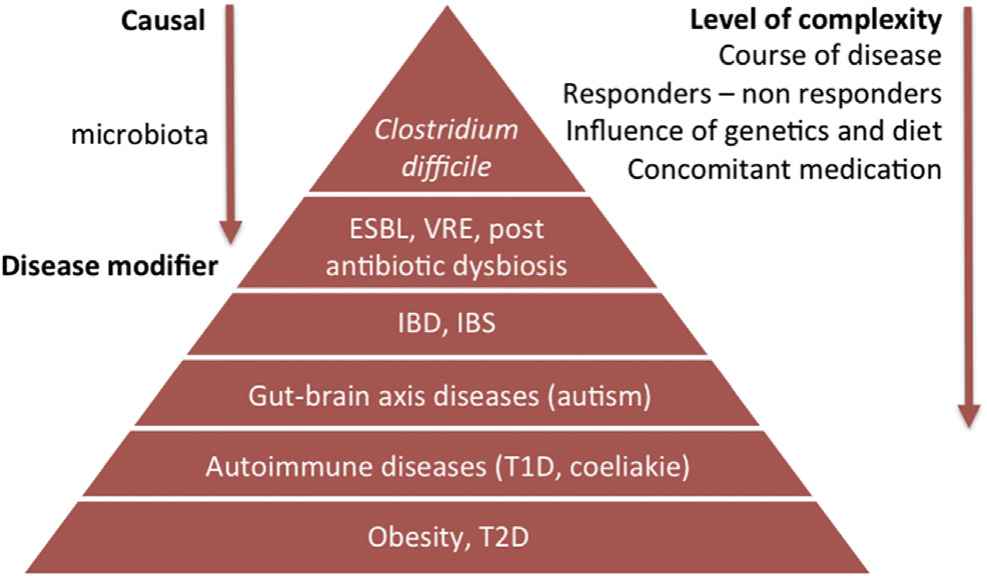
**Different degrees of potential causal relationships between human disease and altered microbiota composition**. The human gut microbiota is a stable ecosystem, which is dependent on environmental and genetic factors. Under healthy conditions, gut microbiota lives in symbiosis with its host. Different human disease states have been associated with altered fecal microbial composition; however, at this moment, it not known whether this is merely a reflection of the underlying disease. Moreover, it is very likely that the intestinal microbiota are not equally important in all human disease states; thus, their role in the pathophysiology may vary from disease modifiers to causal drivers. Abbreviations: ESBL, extended-spectrum beta-lactamase producing Enterobacteriaceae; VRE, vancomycin-resistant enterococci; IBD, inflammatory bowel disease; IBS, irritable bowel syndrome; T1D, Type 1 *diabetes mellitus*; T2D, Type 2 *diabetes mellitus*.

The (gut) microbiota is an organ within an organ (the intestine) that is involved in inflammatory as well as metabolic pathways in the host. Disruption of the gut microbiome may disturb the homeostasis of the microbial ecosystem to an alternative stable state associated with pathophysiological traits in microbiota and host [159]. A higher energy harvest from the microbiota may lead to obesity. An enhanced gut permeability and, subsequently, more bacterial components translocating into the host plasma (endotoxemia) may disturb glucose homeostasis. Induction and promotion of single beneficial bacteria (pre- and probiotics, FMT) may halt or reverse this process. However, at present, it not known whether these intestinal microbiota changes are merely disease modifiers or really causally related to the pathophysiology of several human disease states (Figure 3).

Many other questions remain to be answered. Most work done in the gut microbiota field is done in animals, questioning causality in humans. Further, many contradictory results need to be clarified in future research. However, due to the vast progress in culture-independent methods such as sequencing, it can be expected that our knowledge will rapidly increase and reveal the potential of the (gut) microbiota. We are just beginning to understand how microorganisms influence our health and behavior. Various innovative therapeutic options are in the developing phase with promising outcomes.
